# Plasma Surface Treatment and Application of Polyvinyl Alcohol/Polylactic Acid Electrospun Fibrous Hemostatic Membrane

**DOI:** 10.3390/polym16121635

**Published:** 2024-06-09

**Authors:** Xiaotian Ge, Li Zhang, Xuanhe Wei, Xi Long, Yingchao Han

**Affiliations:** State Key Laboratory of Advanced Technology for Materials Synthesis and Processing, Biomedical Materials and Engineering Research Center of Hubei Province, Wuhan University of Technology, Wuhan 430070, China; 317173@whut.edu.cn (X.G.); zhanglihyc@whut.edu.cn (L.Z.); 336143@whut.edu.cn (X.W.); longxi528702@163.com (X.L.)

**Keywords:** atmospheric pressure cold plasma, polyvinyl alcohol, polylactic acid, surface modification, hemostatic material

## Abstract

In this study, an improved PVA/PLA fibrous hemostatic membrane was prepared by electrospinning technology combined with air plasma modification. The plasma treatment was used to modify PLA to enhance the interlayer bonding between the PVA and PLA fibrous membranes first, then modify the PVA to improve the hemostatic capacity. The surfaces of the PLA and PVA were oxidized after air plasma treatment, the fibrous diameter was reduced, and roughness was increased. Plasma treatment enhanced the interfacial bond strength of PLA/PVA composite fibrous membrane, and PLA acted as a good mechanical support. Plasma-treated PVA/PLA composite membranes showed an increasing liquid-enrichment capacity of 350% and shortened the coagulation time to 258 s. The hemostatic model of the liver showed that the hemostatic ability of plasma-treated PVA/PLA composite membranes was enhanced by 79% compared to untreated PVA membranes, with a slight improvement over commercially available collagen. The results showed that the plasma-treated PVA/PLA fibers were able to achieve more effective hemostasis, which provides a new strategy for improving the hemostatic performance of hemostatic materials.

## 1. Introduction

Massive bleeding caused by surgery or trauma has a high lethality rate, and the use of hemostatic materials to accelerate hemostasis in the early stage of traumatic bleeding is an effective means of saving lives, so research into hemostatic materials with good fluid absorption capacity and wound sealing properties is necessary [1,2,3]. Ideal hemostatic materials should have the abilities of achieving rapid hemostasis, good fluid absorption, strong adhesion to tissues and organs, good biocompatibility, and low cost. Altering the wettability and biocompatibility of materials has been a constant endeavor. With the continuous development of hemostatic materials, many new materials have shown good hemostatic ability. To further develop this, there are also requirements for enhancing the enrichment of coagulation factors in materials to achieve methods of hemostasis [4].

In recent years, researchers have developed many composites that enable rapid hemostasis, such as chitosan, hyaluronic acid, starch, collagen, sodium alginate, hydroxyapatite, and other materials with good biocompatibility. Usually, people add additional hemostatic ability or activate coagulation pathways to enhance the hemostatic efficiency of materials. For example, chitosan can accelerate platelet enrichment, and Ca^2+^ can activate coagulation [5,6,7,8,9]. Meanwhile, some high porosity materials with good infiltration ability can also accelerate coagulation by enriching blood, such as electrospun fibers, MOF, zeolites, and aerogels, etc. [10,11,12]. Polymer materials have shown good performance due to their lower cost and higher biocompatibility. PVA is widely used as a low-cost, non-toxic, easily decomposable material. PVA has excellent hydrophilicity due to the large number of hydroxyl groups, and can be further modified by hydroxyl modification, cross-linking, and oxidization [13]. PVA electrospun fibers in hemostatic applications, due to their limited mechanical properties, have more difficulty facing the complexity of a bleeding situation, particularly in the bleeding of a larger amount, even partially dissolved, so for the PVA fibers membrane, adding a layer of mechanical support is necessary. PLA has good mechanical properties and biocompatibility and is widely used as a low-cost, non-toxic, and easily decomposable material [14]. PVA and PLA can be formed into composite fiber membranes by electrospinning, as an excellent matrix material for tissue repair [15,16,17,18,19,20]. Based on the high porosity and large specific surface area of electrospun fiber membranes, they also display promising applications in hemostasis

Plasma is considered the fourth state of matter and contains many active species, (neutral particles, excited state radicals, photons, electrons, ions, and so on). Plasma surface modification is an efficient, simple, and green modification technology. Plasma surface modification can effectively change the hydrophilicity of materials by introducing hydrophilic functional groups such as –OH, and –COOH on the material surface, and enhance cell adhesion by increasing the surface roughness [21,22,23,24].

In this study, plasma treatment was used to modify PLA to enhance the interlayer bonding between PVA and PLA fibrous membranes first, then modify PVA to improve the liquid-enrichment capacity, resulting in an improved PVA/PLA electrospun fibrous hemostatic membrane. The surface properties (roughness, functional groups, hydrophilicity), tensile modulus, and the hemostatic capacity of fiber membranes were evaluated in a rat liver hemostasis model.

## 2. Materials and Methods

### 2.1. Materials

Polyvinyl alcohol (PVA; Aladdin, 1788; average molecular weight: 20,000–150,000; degree of hydrolysis: 87–89%), polylactic acid (PLA; average molecular weight 980,000, Daigang Bio; Jinan, China), dichloromethane (DCM, Sinopharm; Shanghai, China), N,N′-dimethylformamide (DMF, Sinopharm; Shanghai, China), a cell counting kit (CCK8; HYCEZMBIO; Wuhan, China), and Calcein-AM/PI double stain kit (Yeasen Biotechnology; Shanghai, China).

### 2.2. Preparation of PVA, PLA, and PVA/PLA Fibrous Membranes by Electrospun

The PVA solution was prepared by dissolving 5 g of PVA in 50 mL of deionized water, stirring for 2 h at 80 °C, and then homogenizing for 2 h at room temperature to eliminate air bubbles. The PVA solution was transferred into a 10 mL syringe with a 21 G needle (inner diameter of 0.5 mm). The experiments were carried out at 25 °C and 50% humidity, with an electric field voltage of 11 kV. The pushing rate of the PVA solution was 0.8 ml/h, and the distance from the needle to the receiver was 15 cm. The process lasted for roughly 10 h, to obtain PVA electrospun membranes with a thickness of approximately 0.1 mm.

The PLA solution was prepared by dissolving 0.3 g of PLA in 4 mL of DCM and adding 2 mL of DMF after thorough dissolution. The mass fraction of PLA in DCM was 7.5 wt%. The PLA solution was transferred into a 10 mL syringe with a 20 G needle (inner diameter of 0.6 mm). The experiments were carried out at a temperature of 25 °C and a humidity of 50%, with an electric field of 11 KV. The PLA solution was pushed at a rate of 5 mL/h, and the distance from the needle to the roller receiver was 12 cm, with a roller rotating speed of 1000 r/min. The process lasted for roughly 1 h, to obtain PLA electrospun membranes with a thickness of approximately 0.1 mm.

The PLA electrostatic spinning fibrous membrane was prepared by the above spinning method. Then the PLA fibrous membrane was subjected to plasma treatment for a time set at about 5 min per 2 × 2 cm^2^. After the treatment, the PLA fibrous membrane was re-pasted on tin foil, and the PVA fibers were electrostatically spun on the surface of the PLA fibers by the above-mentioned electrostatic spinning of PVA fibrous membranes. After that, the PVA fiber membrane was plasma treated to obtain the monolayer PVA/PLA composite membrane with a thickness of approximately 0.2 mm.

### 2.3. Plasma Treatment of Fibrous Membranes

Plasma treatment is carried out at atmospheric pressure for a fixed time every 2 × 2 cm^2^. The plasma (RELYON Plasma Piezo Brush PZ3) was operated at a maximum power of 18 W, with power variation settings of 100% (18 W), 80% (14.4 W), 60% (10.8 W), and 40% (7.2 W). The fibrous membrane was placed 0.5 mm below the plasma generator module and treated at different times. Plasma treatment was performed on a non-conductive base.

### 2.4. Characterization of Fibrous Membranes

XPS spectra were acquired on a Thermo Scientific K-Alpha instrument (Waltham, MA, USA) and the acquired data were processed by Advantage software. Aluminum targets are used in XPS testing, scanning starts from energy 1361 eV. FTIR spectra were detected using a Nicolet6700, (Thermo Scientific, Waltham, MA, USA) specification instrument in the range of 400–4000 cm^−1^, with three scans on the same sample. The surface morphology of the fiber membrane was photographed using JSMIT200 (JEOL, Tokyo, Japan), at a voltage of 5 KV, and the fiber diameter was counted using the software ImageJ version 1.51. The process of sample preparation before SEM image shooting included cutting different fiber samples and sticking them onto the sample stage with conductive tape. Finally, after 60 s of gold spraying, the samples were placed in a vacuum chamber for shooting. The number of fiber diameters counted was over 300. AFM images were captured using an atomic force microscope with a Nanoscope Ⅳ specification and the average roughness, Ra, was calculated. AFM scanning size of PVA fiber was 5 μm, and the scanning size of PLA fiber was 10 μm, with a tip size of 20 nm. The contact angle was taken using a Dataphysics OCA20. In the contact angle test, deionized water was used with a droplet size of 50 μL to measure its static contact angle. The contact angle on the surface of PVA fibers was the contact angle formed when the droplet contacted the fiber surface instantly, and the contact angle formed between the droplet and the fiber surface after 8 s. PLA was the contact angle formed when the droplet stabilizes on its surface.

### 2.5. Mechanical Property Test

The mechanical properties of the membranes were tested using an INSTRON 1122 tensiometer. The membranes were cut to a length of 6 cm and a width of 1 cm. The clamping distance was 2 cm, the stretching speed was 10 mm/min, and the stretching was carried out in the length direction. The thickness of the samples was pre-determined using an electronic micrometer and the thickness of the fibrous film was chosen to be around 0.1 mm. The test was repeated five times to calculate the average value and deviation.

### 2.6. Interlayer Bonding

During the plasma treatment of PLA in the preparation process, the PLA electrostatically spun fiber membrane was divided into the plasma-treated area and the untreated area. After electrospinning PVA fibers on its surface, the fibers were cut to the size of 3 × 3 cm^2^ to obtain two kinds of samples, plasma-treated and untreated. The PVA and PLA surfaces were taped separately, and the other end of the tape was pasted on the fixture of the mechanical testing machine to separate the PVA and PLA layers at a tensile speed of 10 mm/min, and the maximum tensile force required for the separation of the two layers was recorded.

### 2.7. Water Absorption

Experiments on the water absorption capacity of fiber membranes were carried out with reference to existing standards [25]. Before the experiment, the fiber was cut to the size of 3 × 3 cm^2^ and after drying, when the fiber membrane was of constant weight, the mass of the fiber membrane was recorded as W_0_. The fiber membrane was placed in deionized water for 24 h and after removing the excess water droplets from the surface of the fiber membrane, the weight was recorded as W_1_ and the water absorption of the fiber membrane was calculated using the following equation:

water absorption (%) = W_1_/W_0_ × 100% (1)

### 2.8. Whole Blood Coagulation Index

Whole blood coagulation experiments were performed based on previous work [26]. Anticoagulated rat whole blood CaCl_2_ (0.2 M) was added 200 μL to each sample, and after 5 min of coagulation, uncoagulated erythrocytes were lysed with deionized water to release hemoglobin, and absorbance was measured at 540 nm. The coagulation index was calculated as follows:

BCI (%) = A_1_/A_0_ × 100% (2)

where A_1_ and A_0_ denote the absorbance of the experimental group and blank control group, respectively.

### 2.9. Clotting Time

In vitro hemostasis experiments were performed using rat anticoagulated blood, which was used within 24 h of collection. For each hemostasis test, fiber membranes of the same size were added to the glass tube, and then 2 mL of rat anticoagulated blood and 80 uL of CaCl_2_ (0.2 M) solution were added, then the mixture was placed at 37 °C. The state of the blood was checked at intervals, and the coagulation time was taken as the time when the glass tube was inverted and no blood flowed.

### 2.10. Hemolysis Assay

The anticoagulated blood containing CaCl_2_ (0.2 M) was diluted 10-fold with saline and mixed with the material (each ml corresponded to a fibrous membrane area of 3 × 3 cm^2^), then the mixed blood was incubated at 37 °C for 4 h. Then, 1 mL of blood was taken and centrifuged at 3000 rpm for 5 min, and the absorbance of 100 μL of supernatant from each sample was measured at 540 nm. The hemolysis rate was calculated as follows:

Hemolysis % = (A_s_ − A_n_)/(A_p_ − A_n_) × 100% (3)

A_s_ is the absorbance of the sample, A_n_ is the absorbance of the positive control (saline), and A_p_ is the absorbance of the negative control (deionized water).

### 2.11. Cytotoxicity

Cytotoxicity was evaluated by means of leachate, which was prepared at 1 mL per 6 × 6 cm^2^ surface area of fibrous membrane. The fibrous membranes were immersed in DMEM medium for 3 days and the temperature was maintained at 37 °C to obtain the extract of the fiber sample. Using the fiber extract for cell culture allows the fiber extract to come into contact with cells to evaluate the cytotoxicity of the material. The subgroups were set as the blank group, plasma-treated PLA group, and treated PVA/PLA composite fibrous group. The experiments were all performed using 24-well plates for cell culture, inoculating cells at a cell concentration of approximately 10^4^ cell/mL, adding 500 μL of the sample extract per well to the well plates, and co-culturing with L929 cells for 1, 3, and 5 days. The blank group was DMEM complete medium. The cell viability was measured by CCK8 kit, the dehydrogenase in the mitochondria of the surviving cells was reduced by WST-8 in the reagent to an orange-colored product; the more the surviving cells, the deeper the orange-colored product is, and the cell viability of different samples can be reflected by the measurement of the absorbance at 450 nm. When the cell viability is above 95%, it indicates that the material has good biocompatibility. The formula for cell viability is as follows:

cell viability = OD_sample_ − OD_0_/OD_control_ − OD_0_ × 100% (4)

OD_sample_, OD_0_, and OD_control_ are the absorbance at 450 nm of the sample, CCK8 solution, and the blank group, respectively.

Cultures of L929 cells were performed in the same way and cells were stained alive and dead with AMPI after 1, 3, and 5 days of culture to determine cell growth.

### 2.12. Liver Hemostasis Experiment

Sprague–Dawley (SD) rats liver laceration model was conducted according to the standard procedure. The SD rats (weight 200 g–300 g) were randomly divided into five groups, the blank control group, untreated PVA group, treated PVA group, commercially available collagen group, and treated PVA/PLA group. Each rat was anesthetized by intraperitoneal injection of 4% *w*/*v* chloral hydrate (0.01 mL/g rat body weight). Rat livers were exposed through an abdominal incision. A bleeding wound (1 cm long and 0.5 cm deep) of the liver was created with a scalpel, and the fibrous membrane was immediately placed on the surface of the wound. Then, the filter paper with adsorbed rat blood was weighed and the amount of bleeding was recorded. All mouse experiments were conducted in accordance with the guidelines of the Laboratory Animal Center of Wuhan University of Technology (Wuhan, China) and approved by the Laboratory Animal Ethics Committee of Wuhan University of Technology. The surviving animals were euthanized at the end of the experiment [1,3]. The percentage reduction in bleeding was calculated as follows:

Percentage reduction in bleeding = (M_0_ − M_1_)/M_0_ × 100% (5)

M_0_ is the blank group and M_1_ is the experimental group.

### 2.13. Statistical Analysis

The results are calculated from three parallel experiments expressed as the mean ± standard deviation. One-way ANOVA was performed to assess significant differences and * *p* < 0.05 was considered statistically significant.

## 3. Results and Discussion

### 3.1. Characterizations of Plasma-Treated PLA

**Figure 1:**
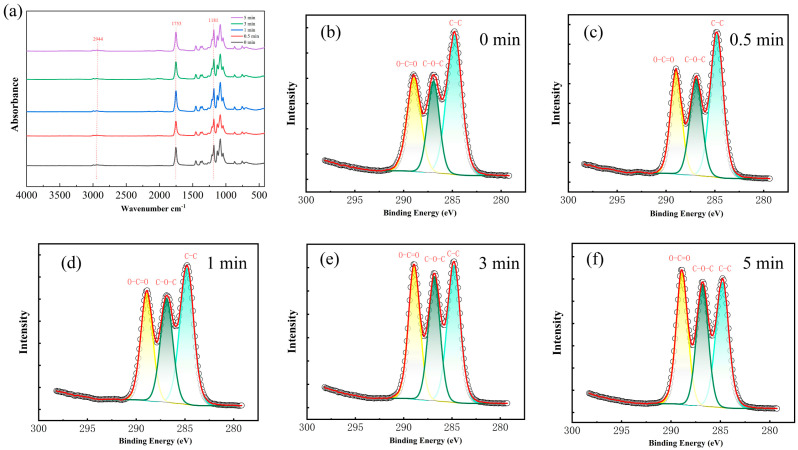
FTIR spectra of PLA with different treatment times (**a**) and C1s fitting with different treatment times of 0 min (**b**), 0.5 min (**c**), 1 min (**d**), 3 min (**e**), 5 min (**f**).

The effect of plasma treatment on the chemical state of atoms on the surface of the fibrous membrane was revealed using XPS. As shown in Figure 1b–f and Supporting Information Tables S1 and S2, three peaks (C–C, C–O, and O–C=O) can be observed at binding energies of 284.8 eV, 286 eV, and 288.5 eV split-fit, respectively [27,28]. It can be seen that the relative content of oxygen on the surface of PLA fibers increased from 34% to 47% and the plasma treatment caused the C–C bonds on the surface of PLA fibers to break continuously from a relative content of 47% to 35%, and the oxygen-containing functional groups (O–C=O, C–O) increased continuously. These results indicate that the air plasma treatment is breaking the C–C bond of the PLA molecular chain and introducing some oxygen-containing functional groups [29].

FTIR spectra further proved the changes in the functional groups on the surface of the fibrous membranes. Some of the classical absorption peaks of PLA can be seen from Figure 1a, with the characteristic carbonyl peak near 1753 cm^−1^ and the C–O–C absorption peak at around 1181 cm^−1^. The weaker hydroxyl absorption peak near 2944 cm^−1^ is due to the fact that the PLA molecular chain has only a small amount of hydroxyl groups at the end [30]. No new functional groups appear in the spectra of the treated fibrous membranes, but their relative contents have changed (Table S3). It was found that the oxygen-containing functional groups on the surface of PLA increased with the treatment time, and the results are generally consistent with XPS.

**Figure 2:**
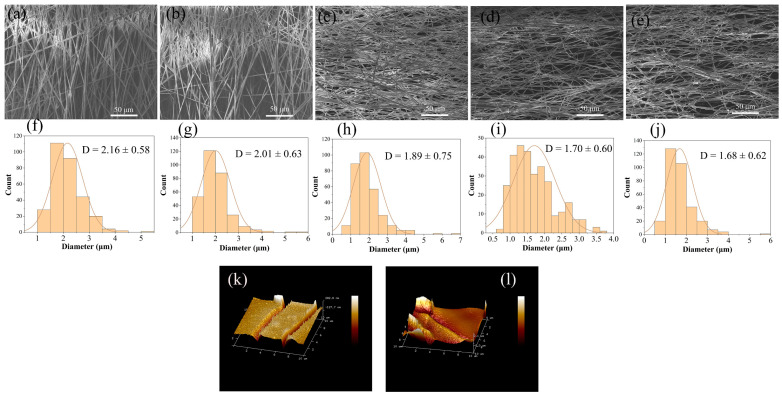
SEM images of PLA electrostatically spun fibrous with different times of plasma treatment and diameter distribution of fibrous (**a**,**f**) 0 min, (**b**,**g**) 0.5 min, (**c**,**h**) 1 min, (**d**,**i**) 3 min, (**e**,**j**) 5 min. (**k**) AFM images of PLA electrostatically spun fibrous membranes without plasma treatment, (**l**) 5 min plasma treatment of the AFM images of PLA electrospun fibrous membranes. The plasma power used was 18 W.

As shown in Figure 2 and Table S4, the diameter of PLA fibers decreased from 2.16 ± 0.58 μm to 1.68 ± 0.62 μm, and the surface roughness gradually increased from 11.27 ± 2.6 nm to 30.73 ± 5.0 nm. The decrease of average diameter and the increase of average roughness can be attributed to the interaction between the plasma and the polymer molecules. The activated high–energy oxygenated plasma strikes the fibers to produce an etching effect [31].

**Figure 3:**
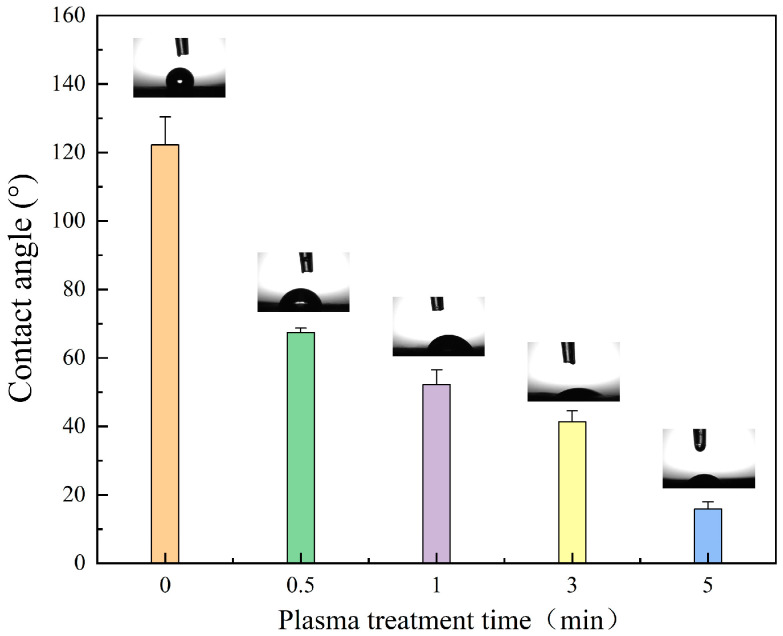
Images of PLA fibrous membranes after different treatment times.

As can be seen in Figure 3, the wettability of PLA changed significantly after plasma treatment, and the contact angle decreased from 122 ± 8.2° to 15 ± 2.1°. There are many factors affecting the wettability of the fiber membrane, and the most important factors affecting PLA should be the increase of polar functional groups on the fiber surface and the microstructure of the fiber surface. From the XPS results, we can see that the increase of oxygen–containing functional groups on the surface of PLA after plasma treatment, which makes the polarity of the fiber surface increase, and the water contact angle on the surface of the fiber with higher polarity is lower. In addition, from the SEM image, we can see that the pore space of the fiber surface has increased, and the liquid droplet infiltrates faster on the surface with higher pore space and rougher surface, which is also one of the reasons for the change in the contact angle of the PLA fibers [28].

### 3.2. Characterizations of Plasma-Treated PVA

**Figure 4:**
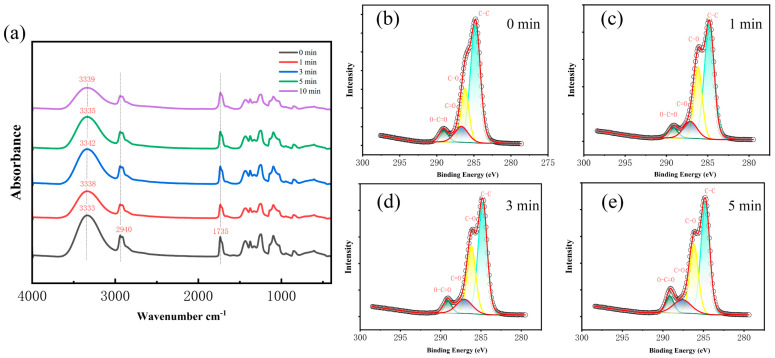
FTIR spectra (**a**) and XPS spectra (**b**–**e**) of PVA fibrous membrane treated by plasma with different processing time.

From Figure 4 and Table S5, it can be seen that the oxygen content on the surface of the PVA fibers membrane is slightly increased, along with the processing time from 0 to 5 min. Moreover, the oxygen content on the surface is slightly increased, along with the rise of the plasma power from 7.2 W to 18 W (Figure S1 and Table S6). Therefore, it can be concluded that both increasing power and extending treatment time are beneficial to raising the content of oxygen on the surface of the fiber membrane. As shown in Figure 2, the four peaks of the split-peak fitting can be observed with binding energies of 284.8 eV, 286 eV, 287.5 eV, and 289 eV, corresponding to C–C, C–O, C=O, and O–C=O, respectively [32]. It can be seen that no new functional groups appear and the relative contents of the functional groups change, as shown in Tables S7 and S8. Results show that increasing power and extending treatment time lead to a decrease in C–C bonds and an increase in C=O, C–OOH bonds, which is generally consistent with the results of other studies [33,34,35]. This implies that plasma treatment generates partial molecular chain breakage and slight carboxylation [36].

As shown in Figure S3, the treated PVA fibrous membranes show typical PVA characteristic peaks of alcohol hydroxyl O–H stretching (3333–3342 cm^−1^), carbonyl C=O stretching (1735 cm^−1^), and C–H stretching (2940 cm^−1^) [37]. The same method was used for the FTIR spectra of PVA, and the analyzed statistics are summarized in Tables S9 and S10. With plasma treatment of 5 min, the increase of power results in the rise of the C=O/C–H ratio, indicating a slight increase in the relative amount of C=O in the molecular chain, which is consistent with the XPS results. Moreover, the O–H/C–H ratio is also decreased, which can be attributed to the oxidation of OH to carboxylate groups. With plasma treatment of 18 W, the increase in treatment time leads to the rise of the C=O/C–H ratio which is consistent with the XPS results. In addition, the ratio of O–H/C–H first increased from 1 min to 3 min, then decreased from 3 min to 5 min. This can be attributed to the interaction between plasma and PVA, involving both the production and oxidation of OH [38]. When the reactive oxygen species generated by plasma treatment are added at different positions on the PVA chain, they correspond to the generation and oxidation of hydroxyl groups. The schematic diagram of possible changes on the PVA chain after plasma treatment is shown in S2. Before 3 min, the production of OH might be the main reaction process and the oxidation reaction process of OH is dominated after 3 min.

**Figure 5:**
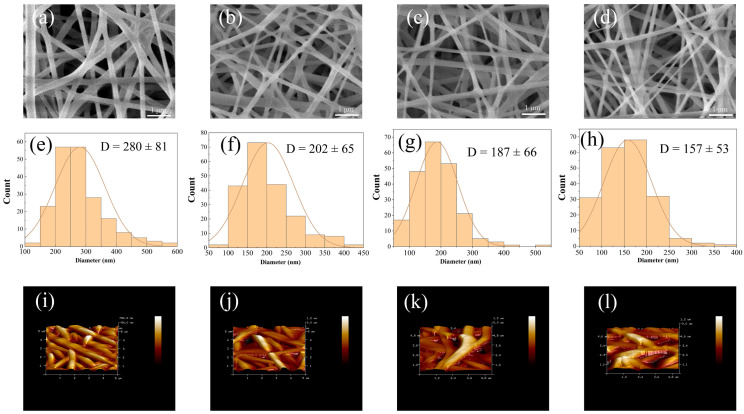
SEM images (**a**–**d**), fiber diameter distribution (**e**–**h**), AFM images (**i**–**l**) of PVA treated by plasma with different times (**a**,**e**,**i**): 0 min, (**b**,**f**,**j**): 1 min, (**c**,**g**,**k**): 3 min, (**d**,**h**,**l**): 5 min.

As shown in Figure 5, along with the rise of plasma treatment time from 0 min to 5 min, the diameter of PVA fibers gradually decreased from 280 ± 81 nm to 157 ± 53 nm, and the surface roughness of PVA fibers gradually increased from 7.38 ± 0.1 nm to 18.8 ± 0.2 nm (Table S11). Similar to the etching on the surface of PLA fibers, plasma etching causes the breakage of PVA molecular chains, which is macroscopically manifested as a gradual decrease in the diameter of PVA fibers.

### 3.3. Wettability

**Figure 6:**
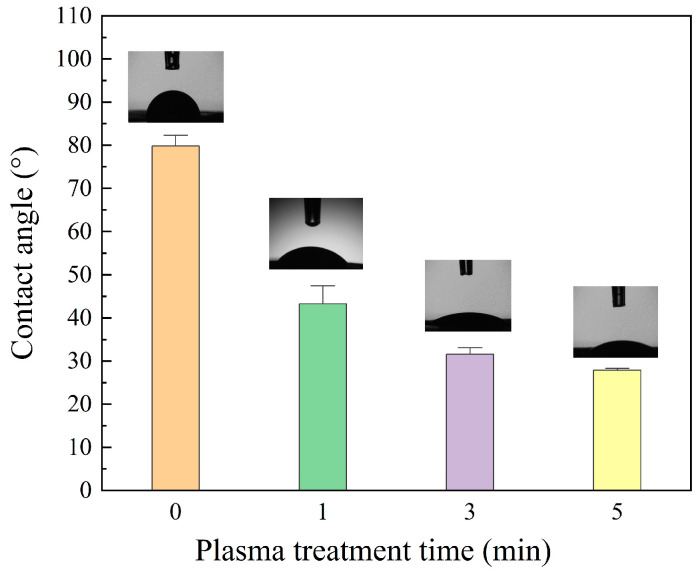
Droplet initial digital photos on PVA fibrous membrane with different treatment time.

After plasma modification, the wettability of the fibrous membrane is significantly improved. As shown in Figure 6, the contact angle drops from 79 ± 3.5° to 29 ± 0.6°, along with the rise of plasma treatment time from 0 min to 5 min. Moreover, the 5 min-treated PVA fibrous membrane shows much faster spreading of droplets than the untreated fibrous membrane, and droplets on 5 min-treated PVA fibrous membrane spread out in less than 8 s. In addition, the moisture absorption experiment proves that the fiber membrane with plasma treatment of 5 min has a faster moisture absorption speed in the early stages. The mesh structure of the fibrous membrane and the roughness, content of polar functional groups, and fiber fraction of fiber surface are the major influencing factors on fibrous membrane wettability [39,40,41,42,43,44]. Before plasma treatment, a large contact angle attributed to the meshes maintains the entrapped air between fibers, decreasing the surface free energy of the PVA fiber membrane. After plasma treatment, the increases in surface roughness and polar functional groups of fibers enhance the wettability of fibrous membranes.

### 3.4. PVA/PLA Tensile Modulus

**Figure 7:**
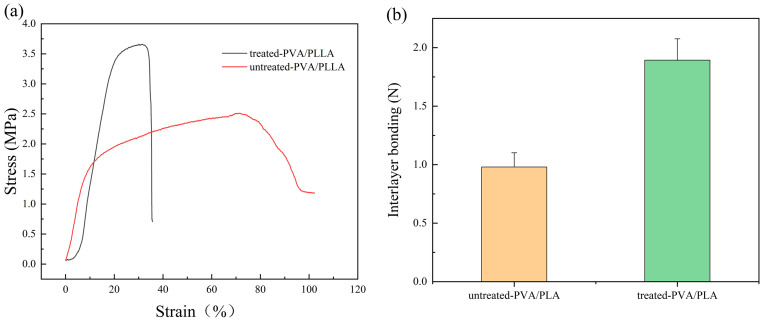
Stress–strain curves (**a**) and interlayer bonding (**b**).

As shown in Figure 7a, the fracture strength of the plasma-treated PVA/PLA fiber membranes increased by about 1 Mpa, but the fracture elongation decreased to some extent, which indicates that the plasma treatment affected the ductility of the PVA/PLA fiber membranes. The mechanical properties of PVA and PLA at different treatment times were tested separately (Figures S4 and S5). It was found that the tensile strain of PVA and PLA fiber membranes decreased and the tensile modulus increased after plasma treatment. In addition, the fracture strength of the treated PVA increased, and the fracture strength of PLA did not change much. The addition of PLA provided some mechanical support for PVA, while the plasma treatment improved the mechanical properties of the fiber membranes with the loss of part of the ductility.

It was reported that air plasma treatment generated the size-related surface effect that the decreasing fiber diameter could increase the tensile modulus dramatically. This is especially evident when the fiber diameter is less than 80 nm. The Young’s modulus of fibrous is calculated by the following equation:

E = k_PVA_ L^3^/48πab^3^ (6)

L is the length of the fibrous in the channel, K_PVA_ is the stiffness of the fibrous; the fibrous is considered as an elliptical cross-section, and a, b are the long and short axes of the ellipse, respectively [45].

In our study, a decrease of fiber diameter of about 40% was observed, so the increase of tensile modulus can be mainly attributed to the decrease in fiber diameter. In addition, other studies showed that plasma treatment could promote the formation of chemical bonds (intermolecular and intramolecular hydrogen) on the surface of the polymer and enhance the cross-linking, leading to an increase in modulus [41].

As shown in Figure 7b, the bonding between the PVA fibers and PLA fibers after plasma treatment increased by about 1 N. The bonding between the fiber membranes before plasma treatment may be due to electrostatic attraction caused by the residual charge of the electrostatic spinning membrane and bonded interlacing between a small number of fibers. The increased probability of bonded interlacing between the two layers of fibers after plasma treatment may be related to the increased roughness of the fiber surface after treatment and the increased void space between the fibers. It has been shown that the stripping energy of PVA fibers from the plasma-treated base increases with the roughness of the base within a certain range [46].

### 3.5. Water Absorption

**Figure 8:**
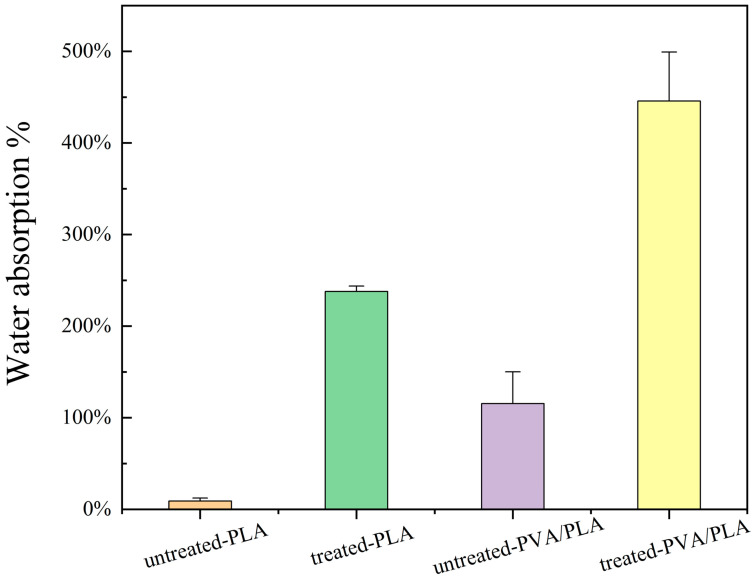
Liquid enrichment capacity of treated and untreated PVA/PLA fiber membranes.

As shown in Figure 8, the water absorption rate of PLA increased to 237%, and the water absorption of a single layer of PVA/PLA increases to about 450% after plasma treatment. The large difference in water absorption before and after plasma treatment is mainly due to the fact that PLA electrospun fibers change from a hydrophobic to a hydrophilic state, while untreated PLA electrospun fibers hardly adsorb water.

### 3.6. Hemostatic Performance Test

The biosafety evaluation was performed before the hemostasis test. From Figures S6 and S7, it can be seen that the hemolysis rate of the treated PVA/PLA composite fibers was less than 5%, and the L929 cells showed normal proliferation, which indicates that the fibers have high biosafety [47].

An in vitro coagulation test was used to assess whether the hemostatic capacity of the treated fibrous membranes was improved. As shown in Figure S8, the BCI of treated PVA fibrous membranes was reduced from 47% to 25%, and the BCI of treated PVA/PLA was reduced to 21%, indicating that the treated fibrous membranes had a greater blood enrichment capacity. In addition, the coagulation time of the treated PVA fibrous membrane was reduced to about 438 s, and the coagulation time of the added treated PLA was reduced to about 258 s. The coagulation time of the treated PVA fibrous membrane was reduced to about 438 s, and the coagulation time of the treated PVA/PLA was reduced to about 258 s. The results can be attributed to the increase in the hydrophilicity of the PVA and PLA fiber membranes and the decrease in the diameter of the fibers, allowing the blood to pass through the surface of the fibrous membranes more rapidly to achieve the enrichment of blood between the fibers, which makes the fibrous membranes more structurally conducive to the enrichment of blood [48].

**Figure 9:**
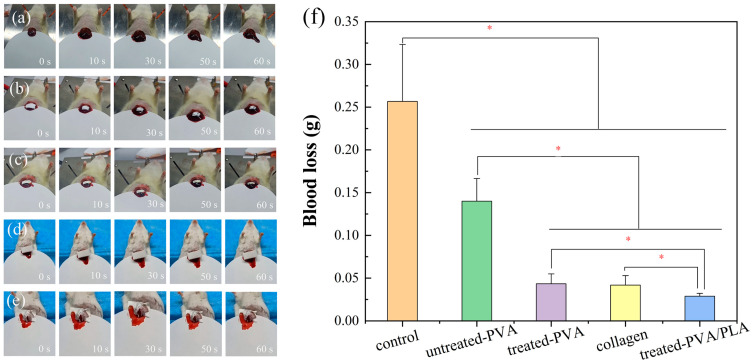
Image of animal experiments with the rat liver hemostasis, (**a**) control, (**b**) PVA, (**c**) plasma, treated PVA, (**d**) commercially available hemostatic collagen, (**e**) treated PVA/PLA, and total blood loss (**f**). * *p* < 0.05 was considered statistically significant.

As shown in Figure 9, in the absence of fibrous membrane hemostasis, the rats exhibited rapid hemorrhage, with a total hemorrhage volume of approximately 0.25 ± 0.07 g in three minutes. Hemostasis was achieved in the PVA group, commercially available collagen group, treated PVA group, and treated PVA/PLA group, with a total hemorrhage of 0.14 ± 0.03 g, 0.0418 ± 0.01 g, 0.04 ± 0.02 g, and 0.029 ± 0.03 g in three minutes, respectively. Bleeding was reduced by 70% and 79% for treated PVA fibers and PVA/PLA fibers, respectively, compared to untreated PVA. The addition of PLA mainly provides mechanical support for the composite membrane, and further enriches the blood to accelerate the coagulation of blood to reduce bleeding. Three-dimensional electrospun fiber membranes with a porous structure have a high absorptive capacity and can concentrate blood to promote coagulation, showing a tamponade effect. Therefore, based on the physical barrier effect, PVA/PLA fibrous membranes show a good hemostatic effect [12,49,50]. Plasma treatment generates the enhancements of wetting properties, mechanical performance, and adhesion capacity of the PVA/PLA fiber membrane, enhancing the physical barrier effect for hemostasis.

## 4. Conclusions

A PVA/PLA hemostatic fiber membrane was prepared using air plasma modification and electrostatic spinning technology. By plasma treatment of PLA surface, the interlayer bonding force between the PVA and PLA fiber membrane increased from 1 N to 2 N. By plasma treatment of PVA, the fracture strength of PVA increased by about 1 MPa, and the amount of liver hemorrhage reduced by about 70%. After the air plasma treatment, the surface oxidation of PLA was somewhat higher than that of PVA, with the oxygen element content elevated by 13% and 2.4%, respectively; the fiber diameters of PLA and PVA were reduced from 2.16 ± 0.58 μm to 1.68 ± 0.62 μm, and 280 ± 81 nm to 157 ± 53 nm, respectively, and the roughness increased from 11.27 ± 2.6 nm to 30.73 ± 5.0 nm and 7.38 ± 0.14 nm to 18.8 ± 0.20 nm. Plasma-treated PVA/PLA composite membranes showed an increasing liquid-enrichment capacity of 350%. The hemostatic model of the liver showed that the hemostatic ability of plasma-treated PVA/PLA composite membranes was enhanced by 79% compared to untreated PVA membranes, and PLA acted as a good mechanical support. PVA/PLA composite membranes therefore have good biocompatibility.

## Data Availability

Data are contained within the article and Supplementary Materials.

## Supplementary Materials

The following supporting information can be downloaded at: https://www.mdpi.com/article/10.3390/polym16121635/s1, Table S1. Plasma treatment of PLA at different times; Table S2. C1s of PLA fibrous membrane with different processing time of plasma treatment (power: 18 W); Table S3. C=O/C–H and C–O/C–H ratios of PLA at different treatment time; Table S4. Surface roughness of PLA electrospun fiber at different treatment times (power: 18 W); Figure S1. XPS spectra of PVA fiber membrane treated by plasma with different powers; Table S5. PVA was treated with plasma at different times with a power of 18 W; Table S6. PVA plasma treatment at different powers for 5 min; Table S7. C1s of PVA fiber membrane with different processing times of plasma treatment (power: 18 W); Table S8. C1s of PVA fibrous membrane with different powers of plasma treatment (time: 5 min); Figure S2. The schematic diagram of possible changes on the PVA chain after plasma treatment; Figure S3. FTIR spectra of PVA fibrous membrane treated by plasma with different powers (a) and different processing times (b); Table S9. Infrared absorbance ratio of O–H/C–H and C=O/C–H at different powers (time: 5 min); Table S10. Infrared absorbance ratio of O–H/C–H and C=O/C–H at different plasma processing times (power: 18 W); Table S11. Surface roughness of fiber at different processing times (power: 18 W); Figure S4. Stress–strain curves (b) and tensile modulus (d) of PLA fibers membrane; Figure S5. Stress–strain curves (b) and tensile modulus (d) of PVA fibers membrane; Figure S6. Hemocompatibility of untreated and treated PVA electrospun fibrous membranes; Figure S7. Cell viability (a) and AMPI staining (b) in blank, treated PLA, and treated PVA/PLA groups for 1, 3, and 5 days; Figure S8. Coagulation index (a), coagulation time (b) of the blank, untreated PVA, treated PVA, and treated PVA/PLA.
