# Supplementary material for: Plasma Surface Treatment and Application of Polyvinyl Alcohol/Polylactic Acid Electrospun Fibrous Hemostatic Membrane
Source: Polymers (Basel). 2024 Jun 9;16(12):1635. doi: 10.3390/polym16121635 (PMC11207798; doi:10.3390/polym16121635)
Supplement: Supplementary file 1 [file polymers-16-01635-s001.zip › polymers-3025615-supplementary.pdf]

[Supporting Information]

Plasma surface treatment and application of polyvinyl alcohol/polylactic acid electrospun fibrous hemostatic membrane

*Xiaotian Ge, Li Zhang, Xuanhe Wei, Xi Long, Yingchao Han**

State Key Laboratory of Advanced Technology for Materials Synthesis and Processing, Biomedical Materials and Engineering Research Center of Hubei Province, Wuhan University of Technology, Wuhan 430070, China

*Corresponding author: Yingchao Han; Address: State Key Laboratory of Advanced Technology for Materials Synthesis and Processing, Biomedical Materials and Engineering Research Center of Hubei Province, Wuhan University of Technology, Wuhan 430070, China.

E-mail: hanyingchao@whut.edu.cn

Table S1. Plasma treatment of PLA at different times.

Plasma exposure time (min)	Element (%)	
	C	O
0	65.23	34.77
0.5	62.83	37.17
1	61.81	38.19
3	59.98	40.02
5	52.39	47.61

Table S2. C1s of PLA fibrous membrane with different processing time of plasma treatment (power:18 W).

XPS-data element	Relative peak area of chemical component (%)					
	Binding energy (eV)	Plasma exposure time (min)				
		0	0.5	1	3	5
C-C	284.8	47.32	44.68	42.38	37.72	35.92
C-O	286	23.76	27.72	28.55	29.95	30.66
O-C=O	288.5	28.92	27.60	29.07	32.32	33.42

Table S3. C=O/C-H and C-O/C-H ratios of PLA at different treatment time.

Plasma exposure time (min)	C=O/C-H	C-O/C-H
0	19.32	18.86
0.5	19.72	18.87
1	19.80	19.00
3	20.38	20.00
5	20.58	20.12

Table S4. Surface roughness of PLLA electrospun fiber at different treatment time (power:18W).

Plasma exposure time (min)	Ra (nm)
0	11.27±2.63
5	30.73±5.00

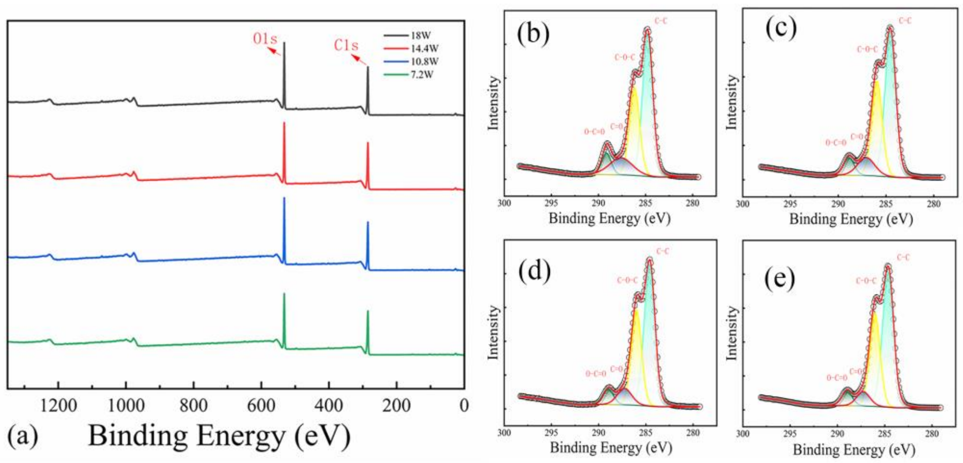

Figure S1. XPS spectra of PVA fiber membrane treated by plasma with different power.

Table S5. PVA was treated with plasma at different times with a power for 18W.

Plasma exposure time (min)	Element	
	C	O
0	71.75	28.25
1	70.81	29.19
3	70.11	29.89
5	69.33	30.67

Table S6. PVA plasma treatment at different powers for 5 minutes.

Different powers (W)	Element	
	C	O
18	69.33	30.67
14.4	69.53	30.47
10.8	69.72	30.28
7.2	70.06	29.94

Table S7. C1s of PVA fiber membrane with different processing time of plasma treatment (power:18 W).

XPS-data element	Relative peak area of chemical component (%)				
	Binding energy (eV)	Plasma exposure time (min)			
		0	1	3	5
C-C	284.8	61.44	54.30	52.39	49.67
C-O	286	27.43	29.25	29.43	30.19
C=O	287.5	5.83	10.48	12.12	13.25
C-OOR、C-OOH	289	5.11	5.97	6.06	6.89

Table S8. C1s of PVA fibrous membrane with different power of plasma treatment (time: 5 min).

XPS-data element	Relative peak area of chemical component (%)				
	Binding energy (eV)	Different powers（W）			
		7.2	10.8	14.4	18
C-C	284.8	53.90	53.02	51.55	49.67
C-O	286	33.87	33.07	32.26	30.19
C=O	287.5	6.68	8.17	10.11	13.25
C-OOR、C-OOH	289	5.54	5.74	6.08	6.89

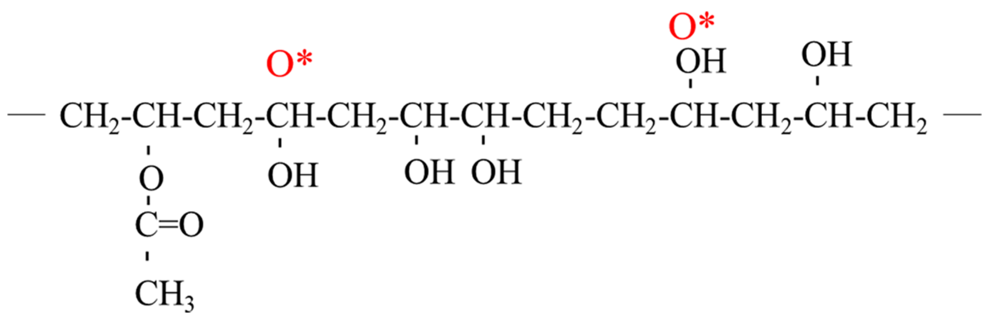

Figure S2. The schematic diagram of possible changes on the PVA chain after plasma treatment.

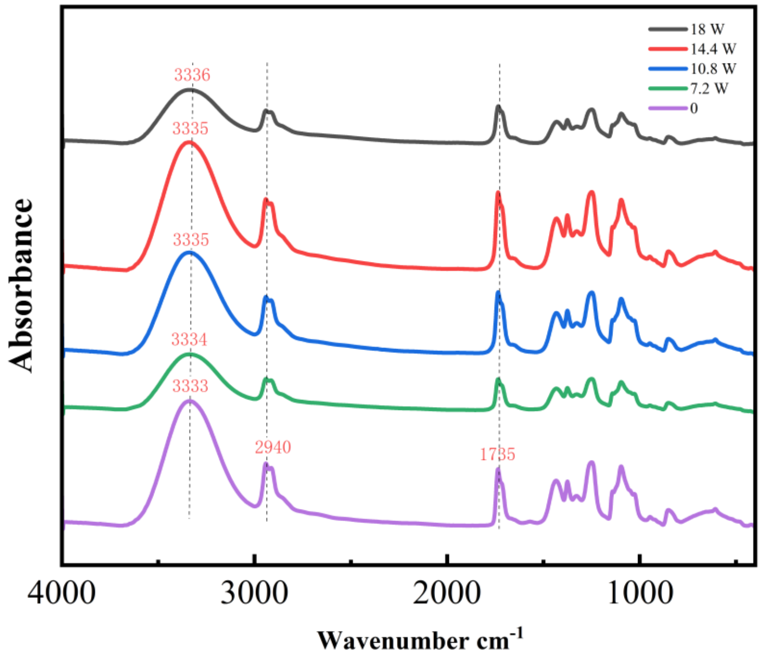

Figure S3. FTIR spectra of PVA fibrous membrane treated by plasma with different power.

Table S9. Infrared absorbance ratio of O-H/C-H and C=O/C-H at different power (time:5 min).

Plasma power（W）	O-H/C-H	C=O/C-H
0	2.600	1.253
7.2	2.414	1.441
10.8	2.393	1.529
14.4	2.387	1.533
18	2.279	1.730

Table S10. Infrared absorbance ratio of O-H/C-H and C=O/C-H at different plasma processing time (power:18W).

Plasma exposure time (min)	O-H/C-H	C=O/C-H
0	2.543	1.227
1	2.603	1.308
3	2.606	1.330
5	2.508	1.351
10	2.279	1.794

Table S11. Surface roughness of fiber at different processing time (power:18W).

Plasma exposure time (min)	Ra (nm)
0	7.38±0.14
1	9.95±0.56
3	11.33±0.25
5	18.8±0.20

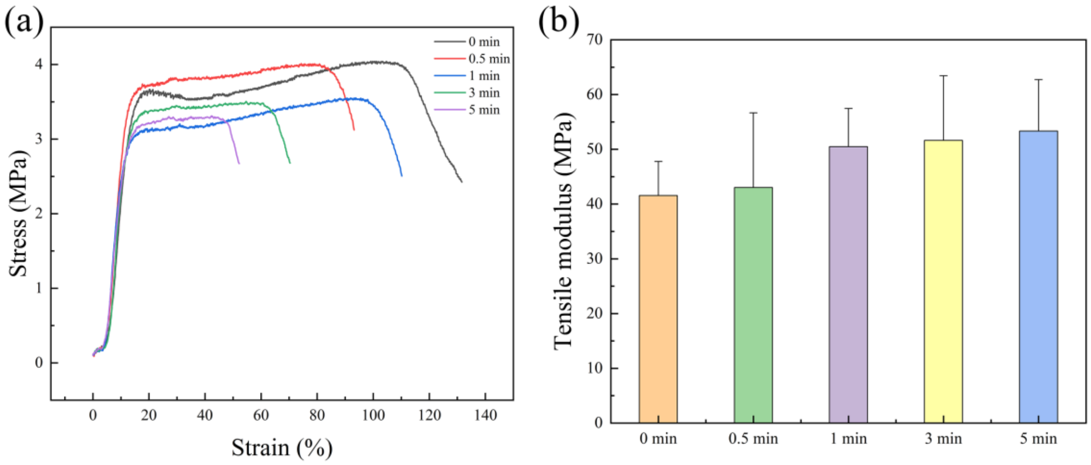

Figure S4. stress-strain curves (b) and tensile modulus (d) of PLA fibers membrane.

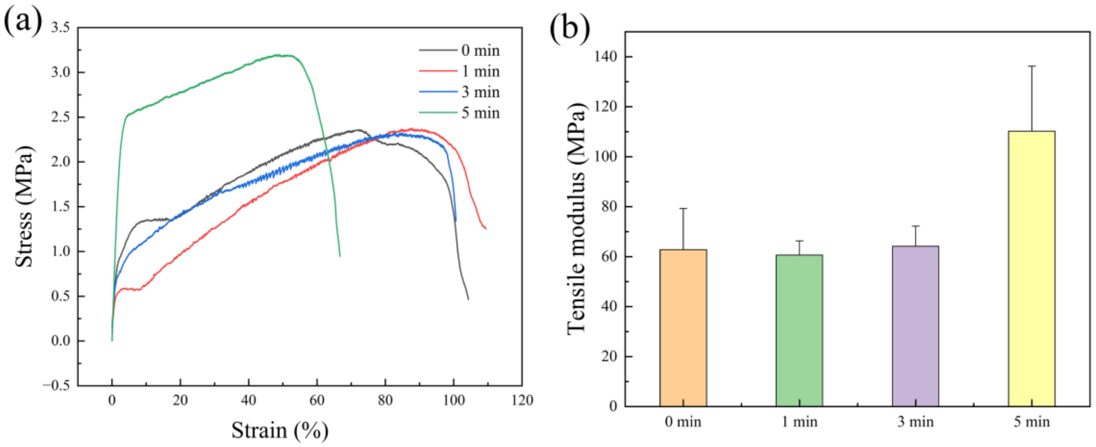

Figure S5. stress-strain curves (b) and tensile modulus (d) of PVA fibers membrane.

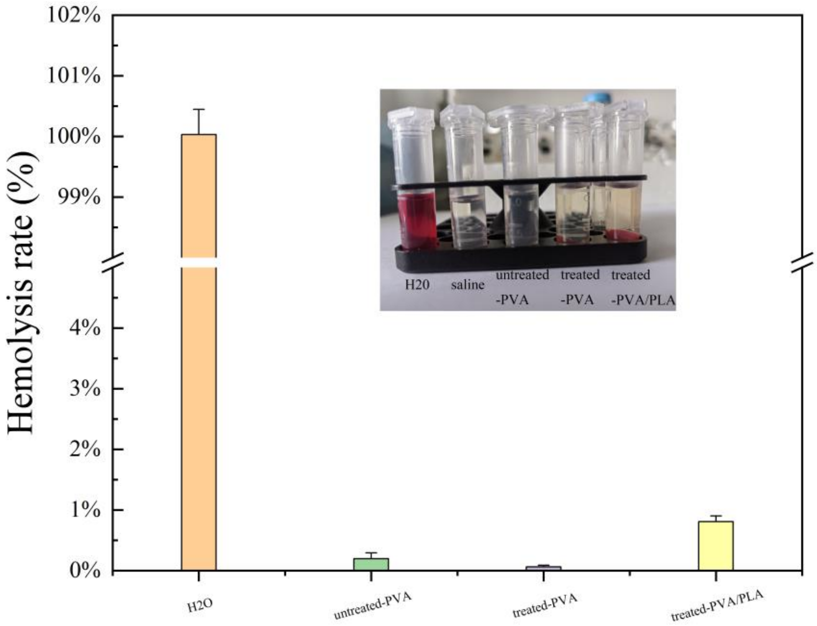

Figure S6. Hemocompatibility of untreated and treated PVA electrospun fibrous membranes

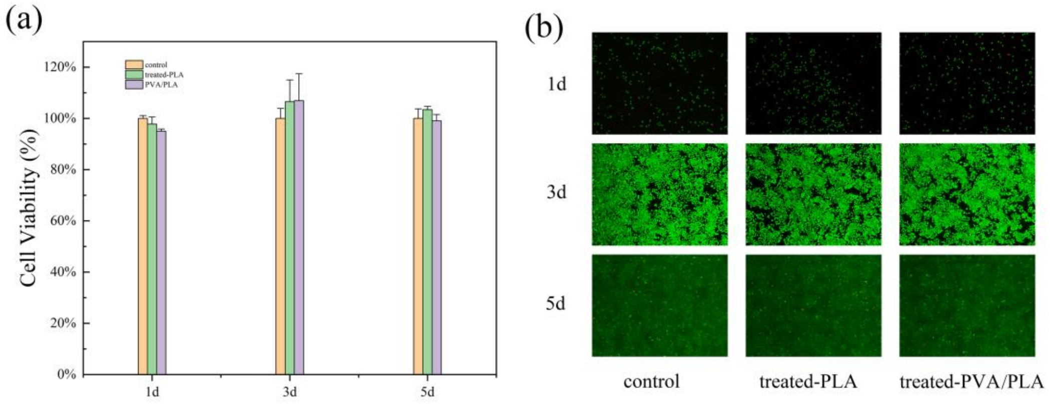

Figure S7. Cell viability (a) and AMPI staining (b) in blank, treated PLA and treated PVA/PLA groups for 1,3,5 days.

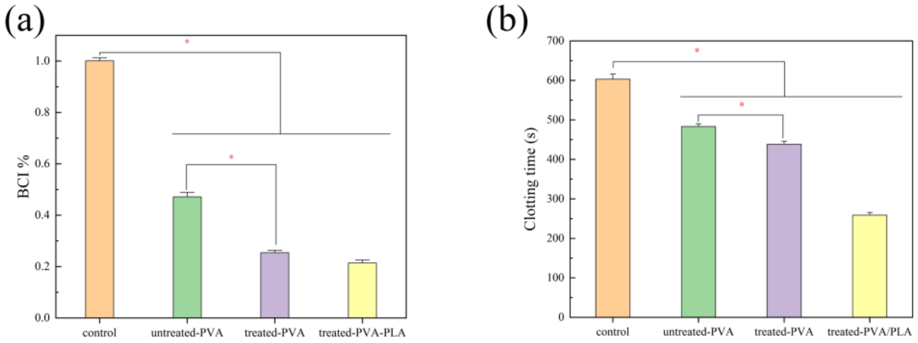

Figure S8. Coagulation index (a), coagulation time (b) of the blank, untreated PVA, treated PVA, and treated-PVA/PLA.
